# Supplementary figures and images for: Nucleotide Sequence Variation in Long-Term Tissue Cultures of Chinese Ginseng (Panax ginseng C. A. Mey.)
Source: Plants (Basel). 2021 Dec 27;11(1):79. doi: 10.3390/plants11010079 (PMC8747682; doi:10.3390/plants11010079)

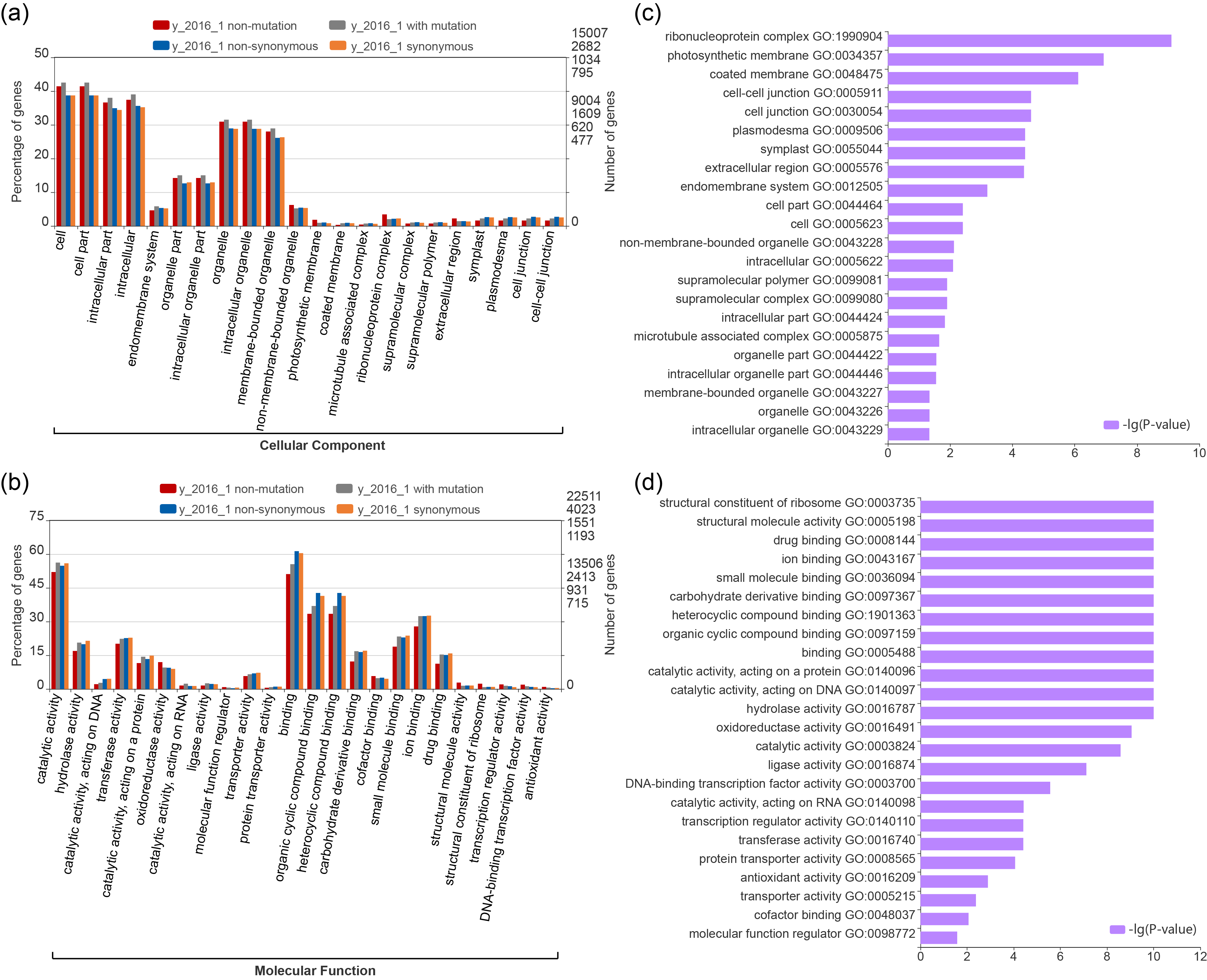

Supplement: Supplementary file 1 [file plants-11-00079-s001.zip › supplementary files/FigureS2a_go_y_2016_1.png]

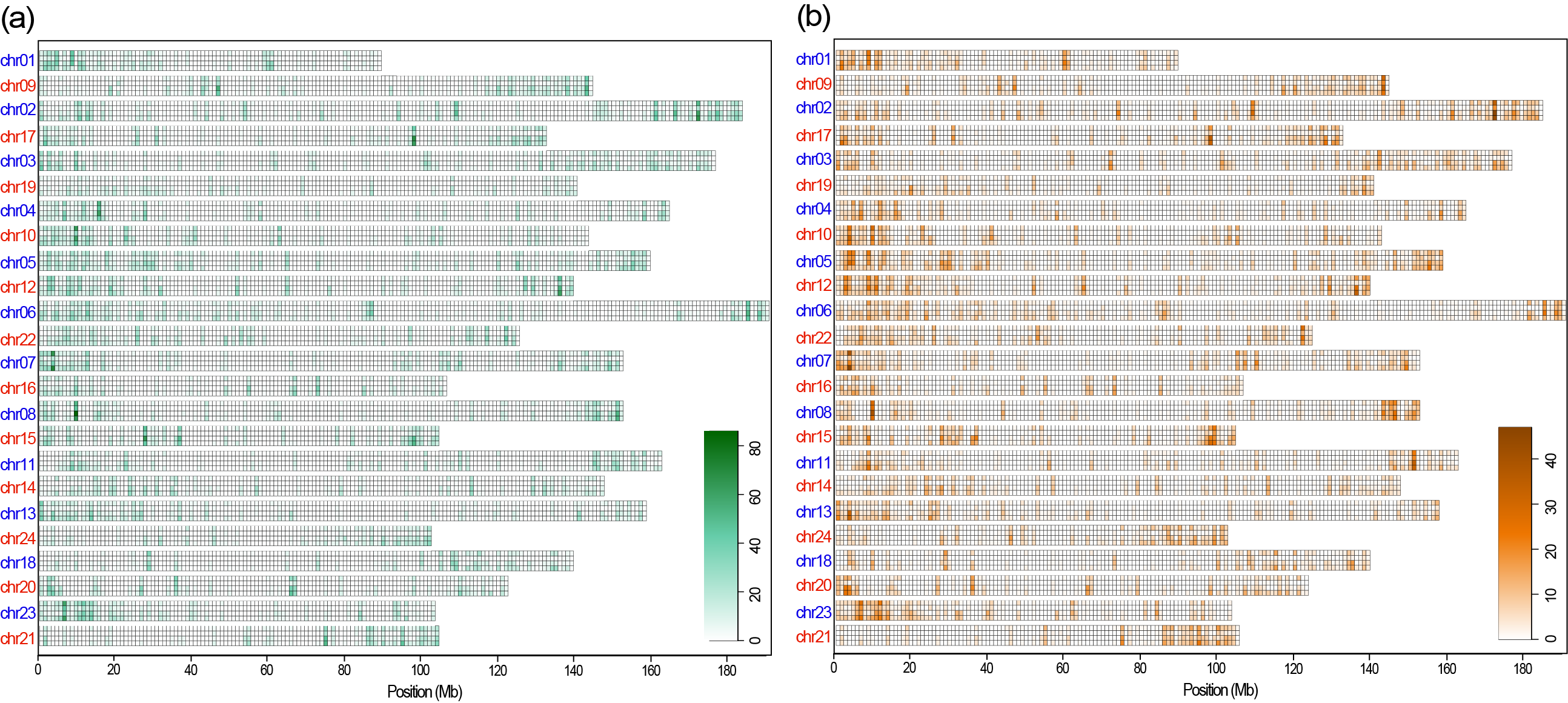

Supplement: Supplementary file 1 [file plants-11-00079-s001.zip › supplementary files/FigureS1_Genomic landscape of non-synonymous and synonymous mutations.png]

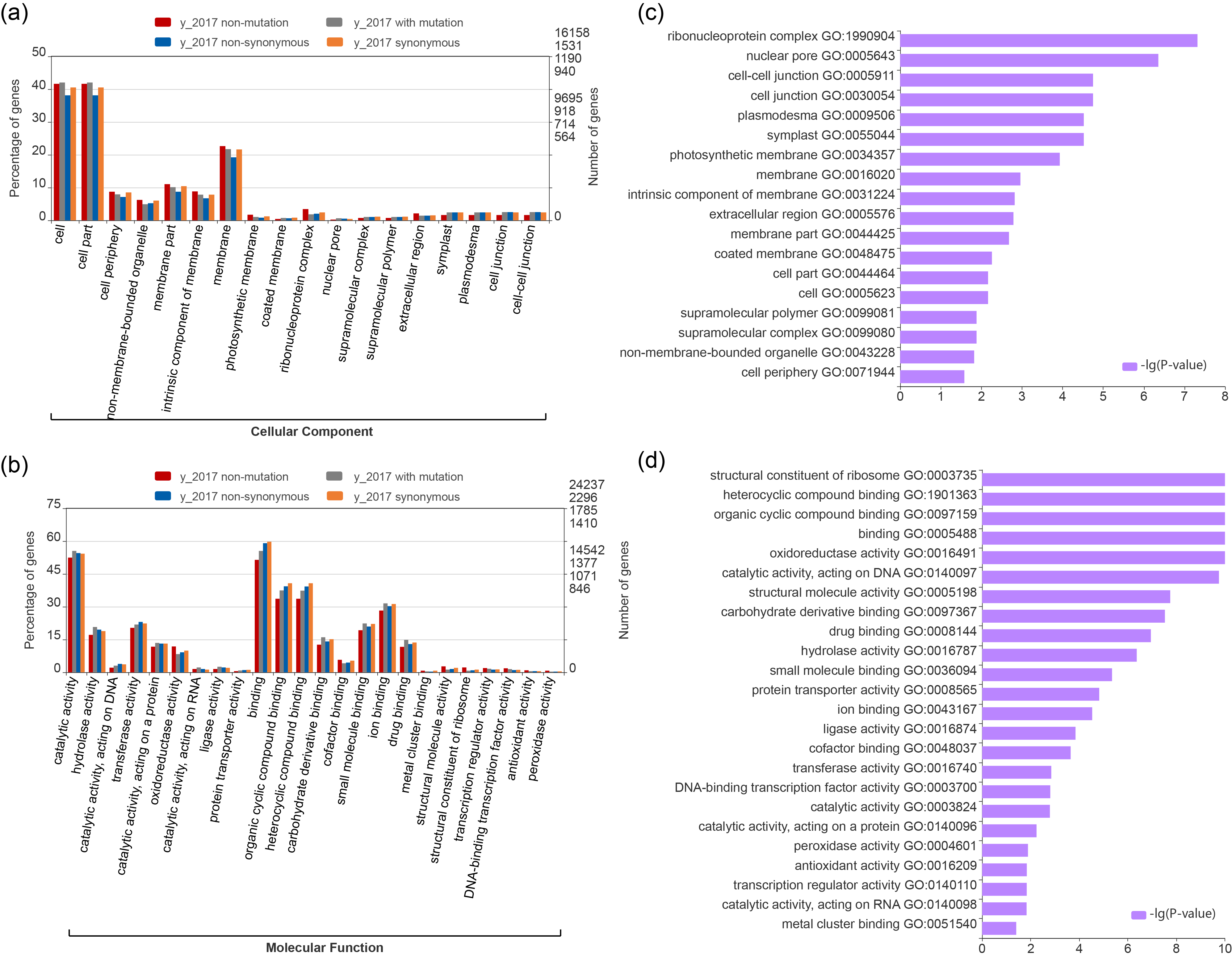

Supplement: Supplementary file 1 [file plants-11-00079-s001.zip › supplementary files/FigureS4a_go_y_2017.png]

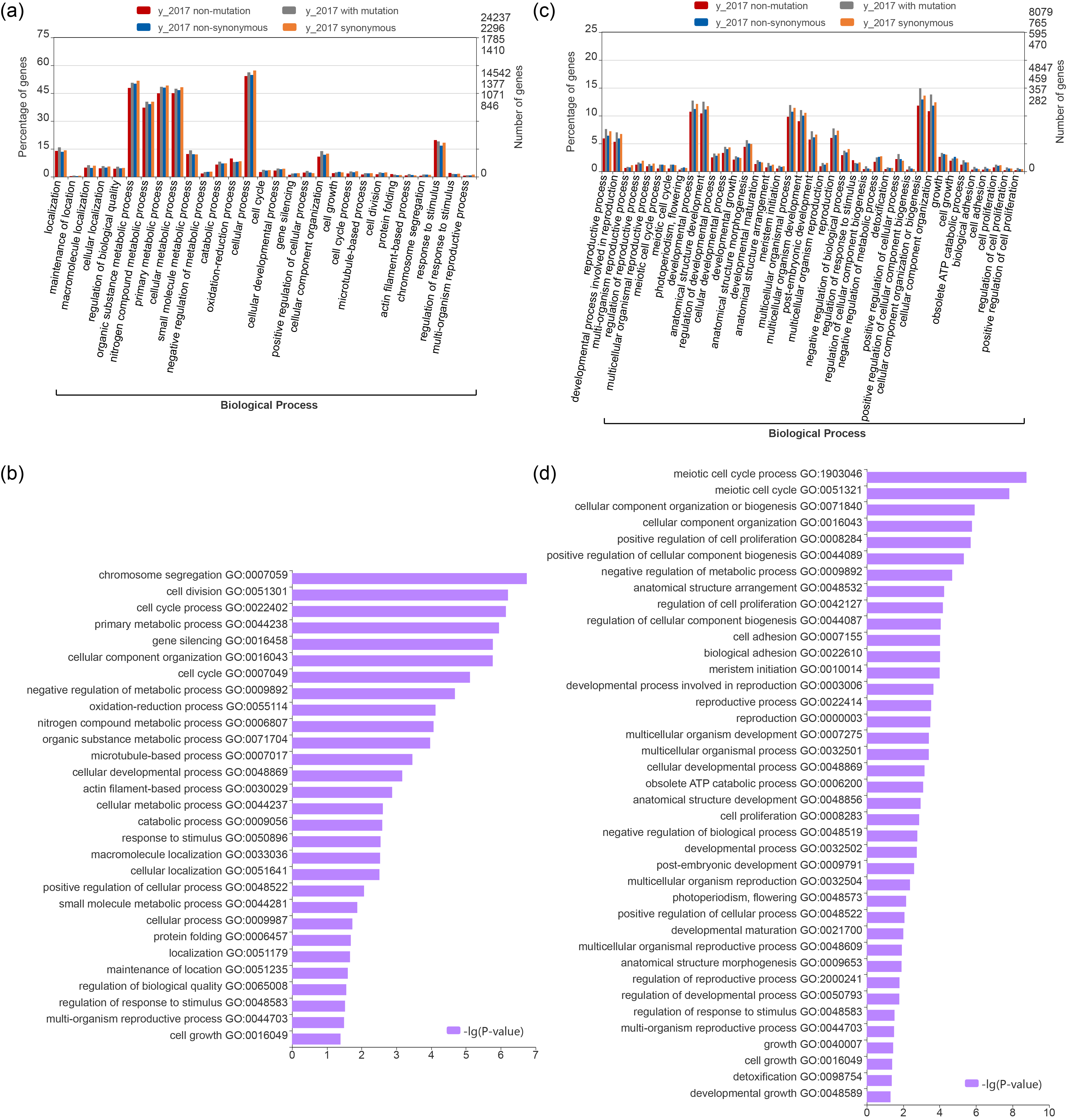

Supplement: Supplementary file 1 [file plants-11-00079-s001.zip › supplementary files/FigureS4b_go_y_2017_part2.png]

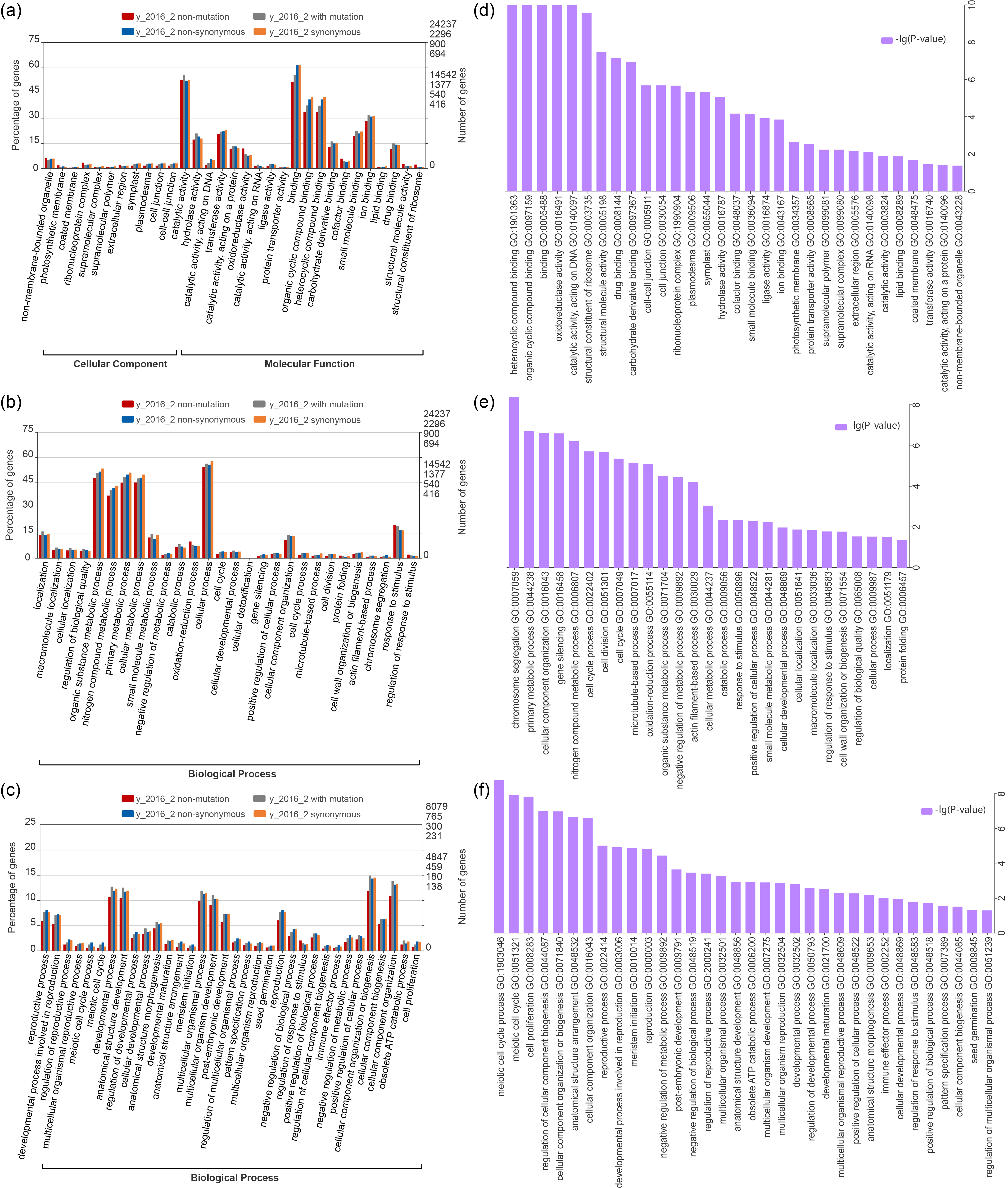

Supplement: Supplementary file 1 [file plants-11-00079-s001.zip › supplementary files/FigureS3_go_y_2016_2.png]

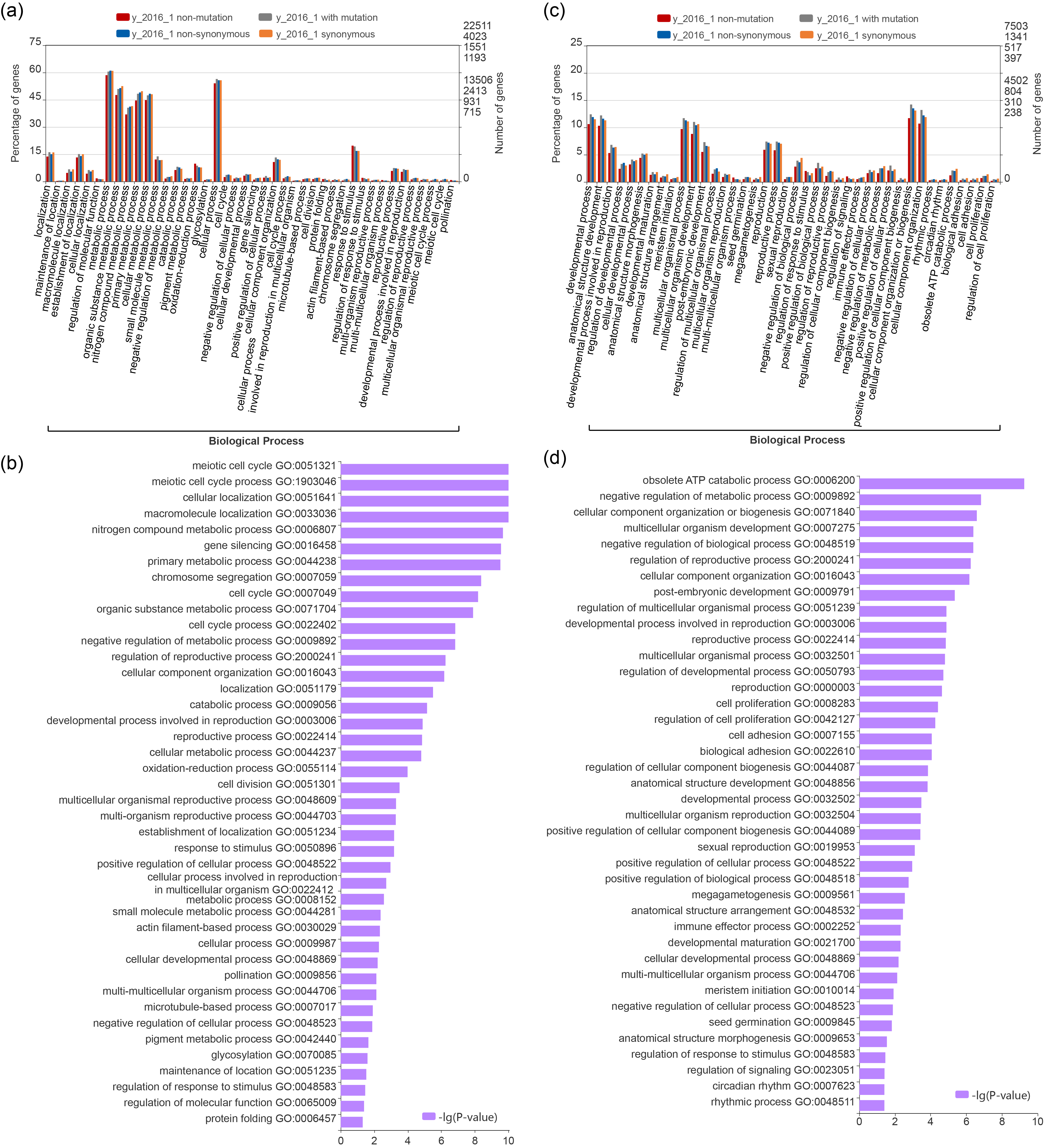

Supplement: Supplementary file 1 [file plants-11-00079-s001.zip › supplementary files/FigureS2b_go_y_2016_1.png]
